# Facile formation of barium titanium oxyhydride on a titanium hydride surface as an ammonia synthesis catalyst†

**DOI:** 10.1039/d3ra01539d

**Published:** 2023-05-22

**Authors:** Yoshihiro Goto, Masashi Kikugawa, Keisuke Kobayashi, Yuichi Manaka, Tetsuya Nanba, Hideyuki Matsumoto, Mitsuru Matsumoto, Masakazu Aoki, Haruo Imagawa

**Affiliations:** a Toyota Central R&D Labs., Inc. 41-1 Yokomichi Nagakute 480-1192 Aichi Japan yoshihiro-goto@mosk.tytlabs.co.jp; b Renewable Energy Research Center, National Institute of Advanced Industrial Science and Technology 2-2-9 Machiikedai Koriyama 963-0298 Fukushima Japan; c Department of Chemical Science and Engineering, School of Materials and Chemical Technology, Tokyo Institute of Technology 2-12-1 Ookayama, Meguro-ku Tokyo 152–8552 Japan

## Abstract

Oxyhydrides are promising compounds as supports for ammonia synthesis catalysts because they suppress hydrogen poisoning on the catalyst surface and enhance the ammonia synthesis activity. Herein, we developed a facile method for preparing BaTiO_2.5_H_0.5_, a perovskite oxyhydride, on a TiH_2_ surface *via* the conventional wet impregnation method using TiH_2_ and Ba hydroxide. Scanning electron microscopy and high-angle annular dark-field scanning transmission electron microscopy observations revealed that BaTiO_2.5_H_0.5_ crystallized as nanoparticles of *ca.* 100–200 nm on the TiH_2_ surface. The Ru-loaded catalyst Ru/BaTiO_2.5_H_0.5_-TiH_2_ exhibited 2.46 times higher ammonia synthesis activity (3.05 mmol-NH_3_ g^−1^ h^−1^ at 400 °C) than the benchmark Ru catalyst Ru–Cs/MgO (1.24 mmol-NH_3_ g^−1^ h^−1^ at 400 °C) because of the suppression of hydrogen poisoning. The analysis of reaction orders showed that the effect of suppressing hydrogen poisoning on Ru/BaTiO_2.5_H_0.5_-TiH_2_ was equivalent to that of the reported Ru/BaTiO_2.5_H_0.5_ catalyst, thus supporting the formation of BaTiO_2.5_H_0.5_ perovskite oxyhydride. This study demonstrated that the selection of appropriate raw materials facilitates the formation of BaTiO_2.5_H_0.5_ oxyhydride nanoparticles on the TiH_2_ surface using the conventional synthesis method.

## Abstract 2

The conventional wet impregnation method yields nanoparticles of BaTi0_2.5_H_0.5_ perovskite hydride on a TiH_2_ surface that works as a support for ammonia synthesis catalysts.

## Graphical abstract

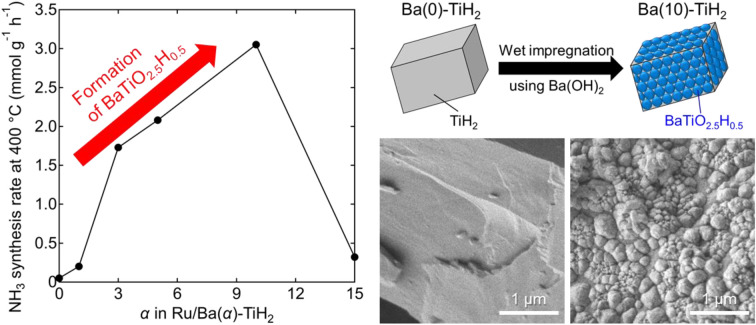

## Introduction

Ammonia (NH_3_), an essential raw material in the production of agricultural fertilizers and synthetic chemicals, has recently attracted attention owing to its applicability as a hydrogen carrier or fuel.^1,2^ Ammonia is predominantly produced *via* the Haber–Bosch (HB) process, which accounts for 1–2% of the global energy demand and 2.5% of global CO_2_ emissions.^3^ Most of the CO_2_ emissions are responsible for hydrogen production processes using steam reforming (CH_4_ + H_2_O → CO + 3H_2_) and water gas shift reactions (CO + H_2_O → CO_2_ + H_2_). Replacing these processes with water electrolysis (2H_2_O → 2H_2_ + O_2_) using renewable energy can significantly reduce this CO_2_ emission.^4^ However, renewable electricity sources of an intermittent nature are not compatible with ammonia synthesis *via* the conventional HB process^5^ because the process is operated on large-scale and steady-state operations. On this basis, ammonia synthesis catalysts need severe reaction conditions (at 450–600 °C and 15–40 MPa).^5^ Therefore, ammonia synthesis catalysts that work under mild conditions should be developed for ammonia synthesis using renewable electricity sources.

The rate-determining step in the synthesis of ammonia (3H_2_ + N_2_ → 2NH_3_) is the dissociation of the N_2_ triple bond (945 KJ mol^−1^), which is the strongest bond among those in diatomic molecules.^6,7^ Supported ruthenium (Ru) catalysts are the most promising candidates for ammonia synthesis under mild conditions because optimum N_2_ adsorption energy facilitates N_2_ dissociation on the Ru surface.^8^ Strongly basic supports (such as CeO_2_, La_0.5_Pr_0.5_O_1.75_, Ba/Ce_0.5_La_0.5_O_1.75_, CeO_2_-PrO_*x*_, and Ce_0.5_La_0.5−*x*_Ti_*x*_O_1.75+0.5*x*_)^9–14^ further promote N_2_ dissociation because the basic compounds enhance the electron transfer from the Ru metal to the antibonding orbital of N_2_.^15^ However, hydrogen atoms generated by H_2_ dissociation are often adsorbed on the active sites of the Ru surface, thereby preventing N_2_ dissociation on the Ru surface.^16^ In recent years, oxyhydrides such as BaTiO_3−*x*_H_*x*_, BaCeO_3−*x*_H_*y*_N_*z*_, LaH_3−2*x*_O_*x*_, GdHO, and SmHO have been reported as supports that suppress hydrogen poisoning and enhance the ammonia synthesis activity of Ru catalysts.^17–20^ Suppression of hydrogen poisoning is presumed to originate from the diffusivity of hydride (H^−^), which allows hydrogen spillover from the Ru metal to the surface of the oxyhydride supports.^21^

Transition-metal (TM) oxyhydride synthesis is generally complicated because the differences in chemical properties (such as reactivity, volatility, and ionic radius) among anions prevent different anions in an identical compound from becoming stable.^22^ For this reason, solid-state topochemical reactions and/or high-pressure reactions have been used to synthesize TM oxyhydrides.^22^ BaTiO_3−*x*_H_*x*_, which is a promising TM perovskite oxyhydride support for ammonia synthesis catalysts, cannot be prepared by simply reducing BaTiO_3_ with hydrogen. However, this oxyhydride is accessible *via* a solid-state topochemical reaction involving BaTiO_3_ and CaH_2_ because the reaction can provide a metastable phase by exchanging the oxide (O^2−^) in BaTiO_3_ with hydride (H^−^) in CaH_2_ while maintaining the basic framework structure of BaTiO_3_.^23^ However, materials preparation using the topochemical reaction is not suitable for practical use because the reaction involves a multi-step process: (1) mixing BaTiO_3_ and the moisture-sensitive CaH_2_ in an inert atmosphere, (2) calcining the mixture for a week under vacuum, and (3) washing in an inert atmosphere to extract product BaTiO_3−*x*_H_*x*_ by removing the residual CaH_2_ and by-product CaO. The practical utility of BaTiO_3−*x*_H_*x*_ may be limited by low producibility owing to the multi-step process. Thus, developing more facile, efficient ways to prepare BaTiO_3−*x*_H_*x*_ is necessary to accelerate the application of BaTiO_3−*x*_H_*x*_. Moreover, the development can lead to the discovery of novel oxyhydrides.

Recently, Uchimura *et al.*^24^ have reported the direct synthesis of BaTiO_3−*x*_H_*x*_ by a mechanochemical method using BaH_2_, BaO, and TiO_2_ and confirmed its performance as a hydrogen-permeable electrode. However, handling in an inert atmosphere is still required because BaH_2_ and BaO are sensitive to moisture. Herein, we demonstrated the synthesis of BaTiO_3−*x*_H_*x*_ (*x* = 0.5), a perovskite oxyhydride, on a TiH_2_ surface *via* conventional wet impregnation method using TiH_2_ and Ba(OH)_2_·8H_2_O, which are stable in moisture and air. The obtained BaTiO_2.5_H_0.5_ was crystallized as fine particles of 100–200 nm size that covered the TiH_2_ particle surface. The Ru-loaded catalyst, Ru/BaTiO_2.5_H_0.5_-TiH_2_, showed 2.46 times higher ammonia synthesis activity than Ru–Cs/MgO as benchmark Ru catalyst. Moreover, Ru/BaTiO_2.5_H_0.5_-TiH_2_ suppressed hydrogen poisoning, thereby proving the formation of BaTiO_2.5_H_0.5_ perovskite oxyhydride.

## Methods

BaTiO_2.5_H_0.5_-TiH_2_ was synthesized *via* the wet impregnation method using TiH_2_ powder and Ba hydroxide solution. TiH_2_ (98%, −325 mesh, Sigma-Aldrich) was impregnated with a solution containing the desired amount of Ba(OH)_2_·8H_2_O (98.0%, FUJIFILM Wako Chemicals) dissolved in 3 : 2 (v/v) H_2_O/ethanol at 230 °C in the air, after which it was heated at 350 °C for 3 h in a 10% H_2_/N_2_ atmosphere. The Ba addition amounts were varied from 0–15 wt% on a Ba(OH)_2_ basis. The obtained compounds are hereafter referred to as Ba(*α*)-TiH_2_ (*α*: wt% of Ba(OH)_2_). Reference compounds Ba(*α*)-TiO_2_ (where *α* = wt% of Ba(OH)_2_) were prepared using the same protocols as for Ba(*α*)-TiH_2_, except TiO_2_ (99.9%, Rutile, Sigma-Aldrich) was used instead of TiH_2_. Ba(10)-TiH_2_ synthesized using Ba(CH_3_COO)_2_ (99.0%, FUJIFILM Wako Chemicals) or Ba(NO_3_)_2_ (99.0%, FUJIFILM Wako Chemicals) instead of Ba(OH)_2_·8H_2_O were also prepared to investigate the effect of Ba sources. Cs/MgO as a benchmark compound was prepared by impregnation using MgO (99%, Sigma-Aldrich) and Cs_2_CO_3_ (99.9%, Sigma-Aldrich)/ethanol solution, followed by thermal treatment at 350 °C for 3 h under 10% H_2_/N_2_. Ru-loaded catalysts, such as Ru/Ba(*α*)-TiH_2_, Ru/Ba(*α*)-TiO_2_, and Ru–Cs/MgO, were prepared by impregnation using Ru_3_(CO)_12_ (99%, Sigma-Aldrich)/tetrahydrofuran solution. The suspension was stirred for 5 h, and the solvent was subsequently evaporated at 27 °C. The obtained compound was dried at 80 °C for 16 h in the air. The Cs and Ru loading amounts were 1 wt% on a metal basis.

X-ray diffraction (XRD) patterns and were collected at room temperature using SmartLab (Rigaku) with Cu Kα radiation (*λ* = 1.54056 Å). The obtained XRD patterns were analyzed using the JANA2006 software.^25^ The neutron diffraction (ND) pattern was collected at room temperature using the NOVA time-of-flight (TOF) neutron diffractometer at the J-PARC facility in Japan. The obtained ND pattern was analyzed using Z-Rietveld software.^26^ Fourier-transform infrared (FT-IR) spectra were recorded using an iS50 spectrometer (Thermo Fisher) equipped with a diffuse reflectance optics accessory. Samples were pretreated at 200 °C for 30 min in flowing He and then examined at 50 °C. Scanning electron microscopy (SEM) images were obtained using a JSM-7400F (JEOL) and a SU3500 (Hitachi) operated at 1.5 kV. High-angle annular dark-field scanning transmission electron microscopy (HAADF-STEM) and energy-dispersive X-ray spectroscopy (EDS) mapping images were obtained using a Tecnai Osiris (FEI) operated at 200 kV. X-ray photoelectron spectroscopy (XPS) was conducted on a Quantera SXM instrument (ULVAC PHI) using Al Kα radiation (1486.6 eV). CO amounts adsorbed by the catalyst were estimated using a BEL-METAL-3 (MicrotracBEL). CO pulse injections (1.99% CO/He) to the samples were conducted at 50 °C after the pre-treatment at 400 °C for 2 h in 100% H_2_.

The ammonia synthesis activities under ambient pressure were estimated using a fixed-bed reactor connected to mass flow controllers. A sample (0.2 g) was suspended on a bed of quartz wool in a quartz tube and preheated at 400 °C for 2 h in 75% H_2_/N_2_ (H_2_/N_2_ = 3) at a flow rate of 80 mL min^−1^. The activity measures were conducted at 300–400 °C in 75% H_2_/N_2_ (H_2_/N_2_ = 3) at a flow rate of 80 mL min^−1^. The ammonia concentrations in the outlet of the quartz tube were monitored by FT-IR spectroscopy and converted into ammonia synthesis rates. Reaction orders of ammonia synthesis with respect to H_2_, N_2_, and NH_3_ were estimated using the method of Aika *et al.*^27^

## Results and discussion

**Fig. 1:**
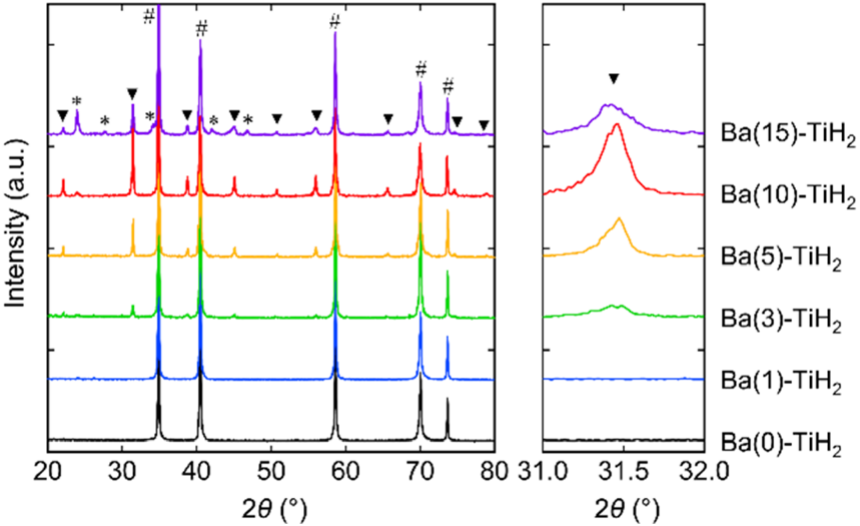
XRD patterns of Ba(*α*)-TiH_2_ (*α* = 0, 1, 3, 5, 10, and 15). Hashtags (#), triangles (▼), and asterisks (*) indicate peaks arising from TiH_2_, BaTiO_2.5_H_0.5_, and BaCO_3_, respectively.

The XRD patterns of Ba(*α*)-TiH_2_ (*α* = 0, 1, 3, 5, 10, and 15) predominantly showed the cubic phase of the fluorite structure (*Fm*3̄*m*) with the lattice parameter of *a* = 4.4489–4.4519 Å (Fig. 1). The lattice parameter matches that of the reported TiH_2_ (*a* = 4.4512(1) Å),^28^ showing that the cubic phase is TiH_2_. The XRD patterns of samples with *α* = 0 and 1 only showed the single phase of TiH_2_, whereas the XRD patterns of *α* ≥ 3 samples contained an additional cubic phase of perovskite structure (*Pm*3̄*m*; Fig. S1 in the ESI†). The lattice volume was estimated to be *V* = 64.926–65.073 Å^3^ (Table 1), which approaches that reported for cubic BaTiO_2.38_H_0.62_ perovskite oxyhydride (*Pm*3̄*m*, *V* = 65.140 Å^3^)^23^ rather than tetragonal BaTiO_3_ perovskite oxide (*P*4*mm*, *V* = 64.281 Å^3^),^29^ which indicates that the additional cubic phase is BaTiO_3−*x*_H_*x*_ perovskite oxyhydride. The H content *x* in BaTiO_3−*x*_H_*x*_ (*α* = 3, 5, 10, and 15) were determined to be *x* = 0.47–0.57 based on Vegard's law (Table 1; hereafter, the obtained perovskite oxyhydride is referred to as BaTiO_2.5_H_0.5_). The formation of oxygen-deficient BaTiO_3−*δ*_ is unlikely because the lattice volume of BaTiO_2.5_H_0.5_ (*V* = 64.926–65.073 Å^3^) is larger than that reported for cubic BaTiO_3−*δ*_ perovskite oxide (*δ* = 0.25, *Pm*3̄*m*, *V* = 64.337 Å^3^) where the lattice volume does not change through the formation of oxygen defects.^30^ The formation of the BaTiO_3−*δ*_(OH)_*δ*_ oxyhydroxide through OH^−^ incorporation is also denied because no peaks associated with OH bonds (observed at 3400 cm^−1^ in BaTiO_3_ system)^31^ were observed by FT-IR spectroscopy (Fig. S2†). Moreover, the XRD patterns of the *α* = 10 and 15 samples showed that BaCO_3_ can potentially form through the reaction of unreacted Ba(OH)_2_ with atmospheric CO_2_ absorbed during impregnation. Thus, the addition of Ba into TiH_2_ forms BaTiO_2.5_H_0.5_ perovskite oxyhydride and BaCO_3_.

**Table 1:** Physical characteristics of Ba(*α*)-TiH_2_ (*α* = 0, 1, 3, 5, 10, and 15)

Sample	Lattice volume of BaTiO_3−*x*_H_*x*_^a^ (Å^3^)	H content *x*^b^
Ba(0)-TiH_2_	—	—
Ba(1)-TiH_2_	—	—
Ba(3)-TiH_2_	65.002(2)	0.52
Ba(5)-TiH_2_	64.926(2)	0.47
Ba(10)-TiH_2_	65.009(2)	0.56
Ba(15)-TiH_2_	65.073(3)	0.57

a Calculated from the lattice parameters based on the cubic perovskite structure (*Pm*3̄*m*, *Z* = 1).

b Determined from Vegard's law using the lattice volumes of BaTiO_3_ (*V* = 64.281 Å^3^)^29^ and BaTiO_2.38_H_0.62_ (*V* = 65.140 Å^3^).^23^

The ND pattern of Ba(10)-TiH_2_ was collected to investigate the presence of the hydride in BaTiO_2.5_H_0.5_ (Fig. S3 and Table S1†). Rietveld refinement was performed by assuming that the secondary phase is a BaTiO_3−*x*_H_*x*_ cubic perovskite with a *Pm*3̄*m* structural model, where Ba, Ti, and O/H atoms are placed at the Wyckoff position of 1*a* (0, 0, 0), 1*b* (0.5, 0.5, 0.5), and 3*c* (0, 0.5, 0.5), respectively. Cubic TiH_2_ (*Fm*3̄*m*) was added as a primary phase. Refinement converged to O and H occupancies of *g*(O) = 0.886(2) and *g*(H) = 0.114(2), which yields the BaTiO_2.658(6)_H_0.342(6)_ composition and supports the notion that perovskite phase contains hydride. We note here that the difference in the neutron scattering lengths of oxygen and hydrogen (O: 5.803 fm, H: −3.741 fm)^32^ gives the composition BaTiO_2.438(6)_ when the refinement is performed by assuming that the secondary phase is oxygen-deficient BaTiO_3−*δ*_. However, because the formation of oxygen-deficient BaTiO_3−*δ*_ is ruled out by considering the lattice volumes estimated by the XRD analysis, the ND results support the formation of an oxyhydride.

**Fig. 2:**
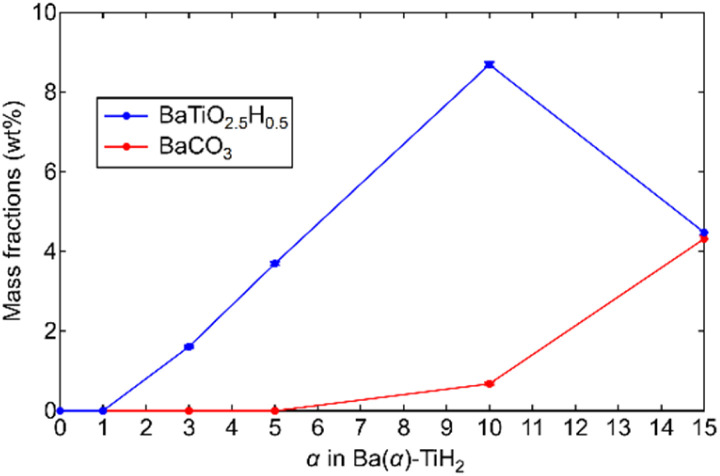
Mass fractions of BaTiO_2.5_H_0.5_ and BaCO_3_ in Ba(*α*)-TiH_2_ determined by Rietveld refinements of the XRD patterns of Ba(*α*)-TiH_2_. Cubic TiH_2_ (*Fm*3̄*m*), cubic BaTiO_2.5_H_0.5_ (*Pm*3̄*m*), and orthorhombic BaCO_3_ (*Pnma*) phases were applied during the analysis.

The degrees of BaTiO_2.5_H_0.5_ and BaCO_3_ formation were determined by Rietveld refinement of the XRD patterns of Ba(*α*)-TiH_2_. We note here that components (less than about 1%) that are not detected in the XRD analysis are not considered. As shown in Fig. 2, the mass fraction of BaTiO_2.5_H_0.5_ is 0% at 0 ≤ *α* ≤ 1 and higher at 3 ≤ *α* ≤ 10 (1.6% to 8.7%). However, a lower fraction was observed at *α* = 15 (4.5%). The addition of Ba to TiH_2_ facilitates the formation of BaTiO_2.5_H_0.5_, while excess Ba inhibits its formation. Thus, the optimum amount of Ba(OH)_2_ needed to form BaTiO_2.5_H_0.5_ corresponds to *α* = 10. The mass fraction of the formed BaCO_3_ was 0% at 0 ≤ *α* ≤ 5 but higher at 10 ≤ *α* ≤ 15 (0.7 to 4.3%), which implies that unreacted Ba(OH)_2_ remains at *α* ≥ 10. The samples also exhibited colors that depend on the amounts of BaTiO_2.5_H_0.5_ and BaCO_3_ amounts (Fig. S4†). Ba(0)-TiH_2_ and Ba(5)-TiH_2_ are gray, which is typical of TiH_2_, while Ba(10)-TiH_2_ is dark blue, which is typical of BaTiO_2.5_H_0.5_,^23^ which also supports the notion that the BaTiO_2.5_H_0.5_ perovskite oxyhydride had formed. Ba(15)-TiH_2_ is brown, which is possibly due to mixed colors associated with TiH_2_, BaTiO_2.5_H_0.5_, and BaCO_3_.

**Fig. 3:**
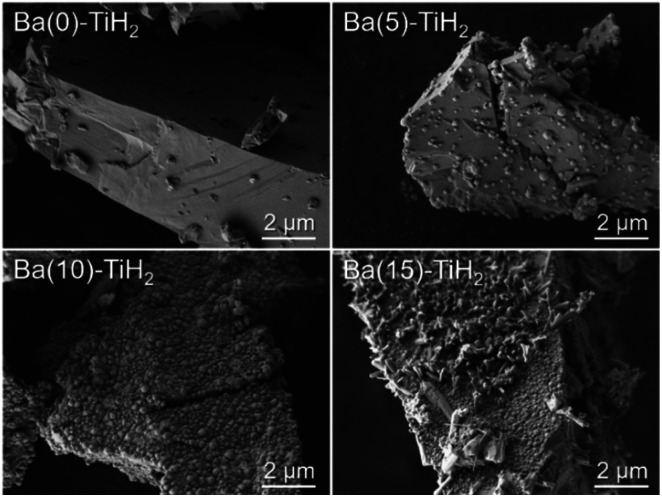
SEM images of Ba(*α*)-TiH_2_ (*α* = 0, 5, 10, and 15) at 100 00× magnification.

**Fig. 4:**
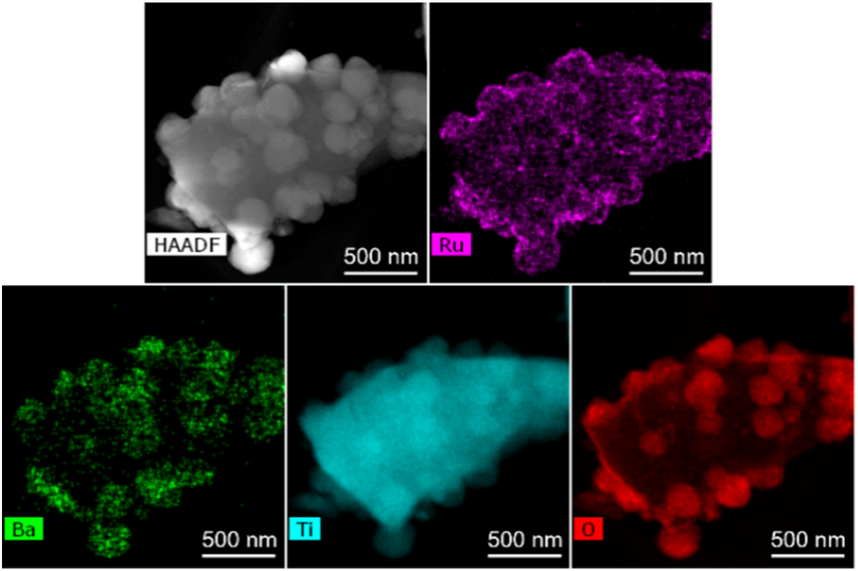
STEM-HAADF and EDX mapping images of Ru/Ba(5)-TiH_2_.

**Fig. 5:**
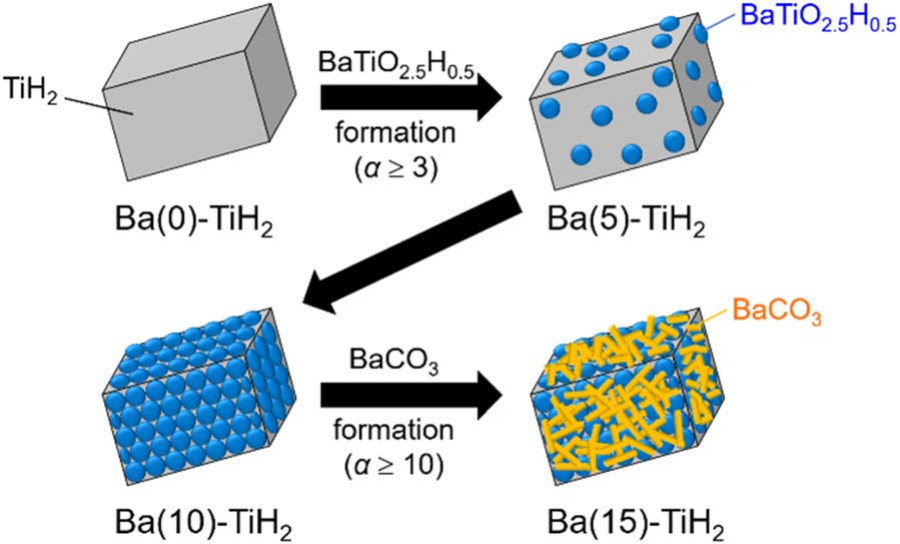
Images of the morphological change of Ba(*α*)-TiH_2_.

The SEM image of Ba(0)-TiH_2_ revealed that the particle size of TiH_2_ ranged from several to tens of μm (Fig. S5 and S6†). Particle size was not affected by the amount of added Ba, which agreed with the identical specific surface area of Ba(*α*)-TiH_2_ (0 ≤ *α* ≤ 15; 1.65–1.91 m^2^ g^−1^). The particle surface of Ba(0)-TiH_2_ and Ba(1)-TiH_2_ were relatively smooth; by comparison, nanoparticles of *ca.* 100–200 nm were dispersed on the TiH_2_ surface of Ba(3)-TiH_2_ and Ba(5)-TiH_2_ (Fig. 3). The nanoparticles were expected to be BaTiO_2.5_H_0.5_ because BaTiO_2.5_H_0.5_ in addition to TiH_2_ was observed in the XRD patterns of Ba(3)-TiH_2_ and Ba(5)-TiH_2_ (Fig. 1). The EDX mappings in the HAADF-STEM image of Ru/Ba(5)-TiH_2_ show that Ba and O elements were localized at the nanoparticles (Fig. 4), thus supporting the identification of the nanoparticles as BaTiO_2.5_H_0.5_. The Ba/Ti atomic ratio estimated from the XPS analysis was higher than that of the feed ratio in the preparation, also supporting that the nanoparticles are BaTiO_2.5_H_0.5_. The SEM image of Ba(10)-TiH_2_ showed that the BaTiO_2.5_H_0.5_ nanoparticles fully covered the TiH_2_ particle. Moreover, the SEM image of Ba(15)-TiH_2_ showed needle-shaped particles on the BaTiO_2.5_H_0.5_ nanoparticles. The crystals were identified as BaCO_3_ according to the XRD pattern because BaCO_3_ generally formed needle-shape crystals.^33^ Therefore, BaTiO_2.5_H_0.5_ was formed as 100–200 nm nanoparticles at *α* ≥ 3, and BaCO_3_ was formed as needle shape particles at *α* ≥ 10 (Fig. 5).

The XRD patterns of Ba(*α*)-TiO_2_ (*α* = 0, 5, 10, 15) synthesized as comparison are shown in Fig. S8.† All samples showed predominantly rutile TiO_2_. Ba(0)-TiO_2_ was the single phase of TiO_2_, whereas Ba(*α*)-TiO_2_ (*α* = 5, 10, and 15) contained additional Ba_2_TiO_4_ and BaCO_3_. The cubic phase of BaTiO_2.5_H_0.5_ formed in Ba(*α*)-TiH_2_ was not observed with the addition of any amount of Ba(OH)_2_. Therefore, TiH_2_ is essential for BaTiO_2.5_H_0.5_ formation.

How is TiH_2_ involved in the formation of BaTiO_2.5_H_0.5_? TiH_2_ consumed to form BaTiO_2.5_H_0.5_ is calculated to be 2.1% for Ba(10)-TiH_2_ which contains the highest amount of BaTiO_2.5_H_0.5_ (8.7%; Fig. 2), suggesting that only the surface part of the TiH_2_ particles contributes to the formation. This is supported by the SEM and TEM observations (Fig. 3 and 4). Because metal hydrides are generally unstable in an oxidizing atmosphere, the surface of TiH_2_ particles is known to be covered by an oxide film of TiO_2_.^34^ TiH_2_ (Ti^2+^) partially oxidized to TiO_2_ (Ti^4+^) possibly contributes to the formation of BaTiO_2.5_H_0.5_ (Ti^3.5+^) *via* the reaction Ba(OH)_2_ + 0.5*x*TiH_2_ + (1 − 0.5*x*)TiO_2_ → BaTiO_3−*x*_H_*x*_ + H_2_O. Moreover, the effect of Ba reagents on BaTiO_2.5_H_0.5_ formation was investigated. The XRD patterns of Ba(10)-TiH_2_ synthesized using Ba(CH_3_COO)_2_ or Ba(NO_3_)_2_ instead of Ba(OH)_2_·8H_2_O are shown in Fig. S9.† The XRD pattern of Ba(10)-TiH_2_ synthesized *via* Ba(CH_3_COO)_2_ showed only TiH_2_ and Ba(CH_3_COO)_2_ and no formation of BaTiO_2.5_H_0.5_. While Ba(10)-TiH_2_ synthesized *via* Ba(NO_3_)_2_ contained BaTiO_2.5_H_0.5_, its mass fraction (1.5%) was only 0.17-times that of the Ba(10)-TiH_2_ synthesized *via* Ba(OH)_2_·8H_2_O (8.7%). These observations suggest that the hydroxide ion (OH^−^) promotes the formation of BaTiO_2.5_H_0.5_.

**Fig. 6:**
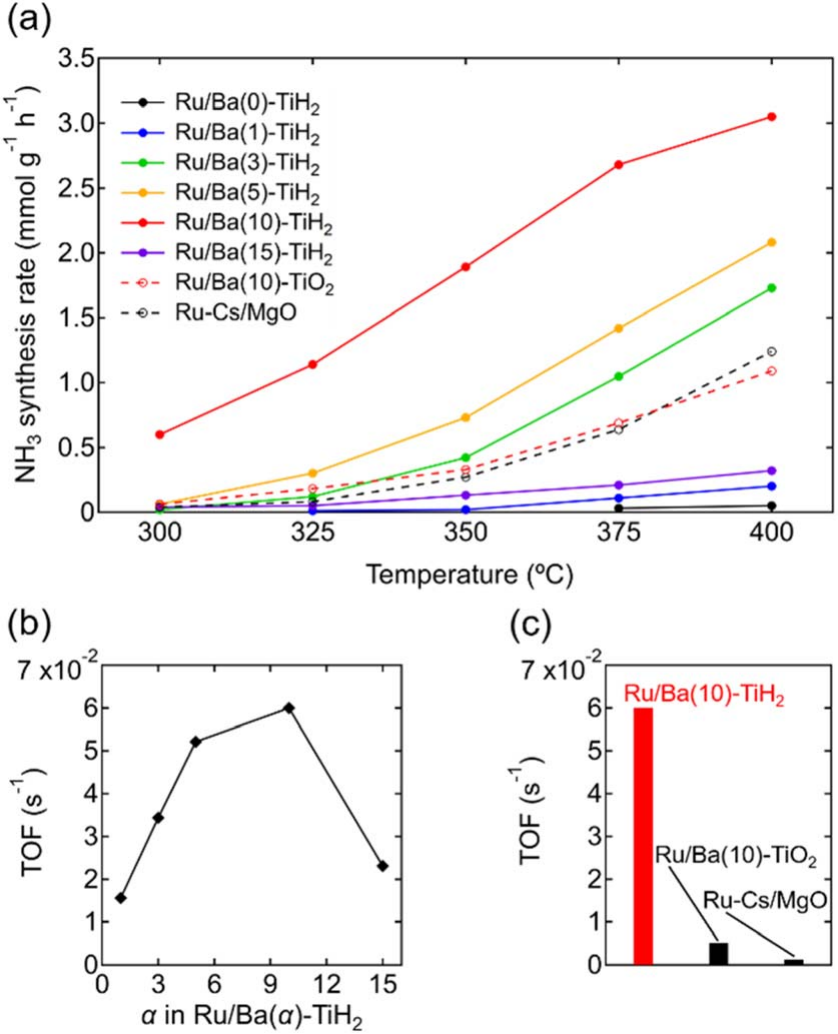
(a) Temperature dependence of the NH_3_ synthesis rates for Ru/Ba(*α*)-TiH_2_ (*α* = 0, 1, 3, 5, 10, and 15), Ru/Ba(10)-TiO_2_, and Ru–Cs/MgO (reaction conditions: catalyst, 0.2 g; reaction gas, H_2_/N_2_ = 3 at a flow rate of 80 mL min^−1^; pressure = ambient pressure). (b) TOF of Ru/Ba(*α*)-TiH_2_ (*α* = 0, 1, 3, 5, 10, and 15) at 350 °C as functions of Ba(OH)_2_ added amount *α*. (c) TOF of Ru/Ba(10)-TiH_2_, Ru/Ba(10)-TiO_2_, and Ru–Cs/MgO at 350 °C.

The ammonia synthesis activities of Ru/Ba(*α*)-TiH_2_ (0 ≤ *α* ≤ 15) catalysts were examined at 300–400 °C under ambient pressure (Fig. 6a). Ammonia synthesis rates of all catalysts increased at elevated temperature. Ru/Ba(0)-TiH_2_ exhibited ammonia synthesis activity at ≥375 °C. The ammonia synthesis rate of Ru/Ba(0)-TiH_2_ (0.05 mmol g^−1^ h^−1^ at 400 °C) was 0.04 times that of Ru/Cs–MgO (1.24 mmol g^−1^ h^−1^ at 400 °C), which was often called as benchmark Ru catalysts.^35,36^ The activity of Ru/Ba(1)-TiH_2_ (0.20 mmol g^−1^ h^−1^ at 400 °C) was higher than that of Ru/Ba(0)-TiH_2_ but remained lower than that of Ru/Cs–MgO. However, by contrast, the activities of Ru/Ba(3)-TiH_2_, Ru/Ba(5)-TiH_2_, and Ru/Ba(10)-TiH_2_ (1.73, 2.08, and 3.05 mmol g^−1^ h^−1^ at 400 °C, respectively) were higher than that of Ru–Cs/MgO by a factor of 1.40, 1.68, and 2.46, respectively. Activity increased with the increase in Ba addition amount in 0 ≤ *α* ≤ 10, whereas the activity decreased at *α* = 15 (0.32 mmol g^−1^ h^−1^ at 400 °C). Therefore, the most active catalyst among Ru/Ba(*α*)-TiH_2_ (0 ≤ *α* ≤ 15) was Ru/Ba(10)-TiH_2_. The activity of Ru/Ba(10)-TiH_2_ was higher than that of Ru/Ba(10)-TiO_2_ (1.09 mmol g^−1^ h^−1^ at 400 °C), thereby suggesting that BaTiO_2.5_H_0.5_ formation was responsible for the high activity of Ru/Ba(10)-TiH_2_.

The TOF for the ammonia synthesis reaction at 350 °C was estimated to obtain a deeper insight into the correlation between ammonia synthesis activity and catalyst composition. As shown in Fig. 6b, the TOF of Ru/Ba(*α*)-TiH_2_ increased with increasing amount of Ba addition at 1 ≤ *α* ≤ 10 (1.56 × 10^−2^ s^−1^ to 6.00 × 10^−2^ s^−1^) but decreased at *α* = 15 (2.31 × 10^−2^ s^−1^). This trend was consistent with the trend of the mass fraction of BaTiO_2.5_H_0.5_ (Fig. 2); these observations support that BaTiO_2.5_H_0.5_ formation contributes to the increase in ammonia synthesis activity. This was supported by the fact that the TOF of Ru/Ba(10)-TiH_2_ (6.00 × 10^−2^ s^−1^) was 12 and 46 times larger than those of Ru/Ba(10)-TiO_2_ (0.51 × 10^−2^ s^−1^) and Ru–Cs/MgO (0.13 × 10^−2^ s^−1^), respectively (Fig. 6c). Interestingly, the TOF of Ru/Ba(15)-TiH_2_ (2.31 × 10^−2^ s^−1^) was lower than that of Ru/Ba(5)-TiH_2_ (5.21 × 10^−2^ s^−1^) despite the higher mass fraction of BaTiO_2.5_H_0.5_ for Ru/Ba(15)-TiH_2_ (4.48%) than that for Ru/Ba(5)-TiH_2_ (3.70%). The formation of BaCO_3_, which partially covered the BaTiO_2.5_H_0.5_ particles (Fig. 3), was expected to inhibit the ammonia synthesis reaction of Ru/Ba(15)-TiH_2_ because BaCO_3_ was stable in the reaction temperature.^37^

Finally, reaction orders with respect to H_2_, N_2_ and NH_3_ were investigated using the method of Aika *et al.*^27^ (Fig. S10 and Table S3†). The H_2_ order of Ru/Ba(10)-TiH_2_ (0.15) was higher than that of Ru–Cs/MgO (−0.59), reflecting that compared to Ru–Cs/MgO, Ru/Ba(10)-TiH_2_ had less hydrogen poisoning, which prevented N_2_ dissociation on Ru (Table 2).^16^ The low hydrogen poisoning effect allowed Ru/Ba(10)-TiH_2_ to exhibit higher ammonia synthesis activity than Ru–Cs/MgO. The N_2_ orders of Ru/Ba(10)-TiH_2_ (0.79) and Ru–Cs/MgO (0.89) were virtually coincident, reflecting that the rate-determining step for both catalysts was the unimolecular cleavage reaction of N_2_ for both catalysts. Moreover, the orders of Ru/Ba(10)-TiH_2_ (H_2_ order: 0.15, N_2_ order: 0.79) agreed well with the reported orders of Ru/BaTiO_2.5_H_0.5_ (H_2_ order: 0.2, N_2_ order: 0.7).^17^ Because Ru/BaTiO_3_, the reference catalyst for Ru/BaTiO_2.5_H_0.5_, exhibits stronger hydrogen poisoning due to the absence of hydride in the support perovskite (H_2_ order: −0.89, N_2_ order: 1.2),^17^ the agreement of the H_2_ and N_2_ orders between Ru/Ba(10)-TiH_2_ and Ru/BaTiO_2.5_H_0.5_ supports the formation of BaTiO_2.5_H_0.5_ in Ru/Ba(10)-TiH_2_. The NH_3_ order of Ru/Ba(10)-TiH_2_ (−0.36) was higher than that reported for Ru/BaTiO_2.5_H_0.5_ (−0.64), indicating that the ammonia decomposition reaction on Ru/Ba(10)-TiH_2_ was relatively inhibited. The inhibition may be attributed to the reaction pressure of this study (ambient pressure) being lower than that of reported Ru/BaTiO_2.5_H_0.5_ (5 MPa) because the ammonia decomposition reaction generally proceeded at a higher pressure.^38^

**Table 2:** Reaction orders^a^ for ammonia synthesis reaction over Ru/Ba(10)-TiH_2_ and Ru–Cs/MgO

Catalyst	Order		
	H_2_	N_2_	NH_3_
Ru/Ba(10)-TiH_2_	0.15	0.79	−0.36
Ru–Cs/MgO	−0.59	0.89	0.11

a Estimated from results of kinetic analysis shown in Fig. S8.

## Conclusions

The selection of appropriate raw materials allowed for BaTiO_2.5_H_0.5_ perovskite oxyhydride nanoparticles to be formed on the TiH_2_ surface through the conventional wet impregnation method. BaTiO_2.5_H_0.5_ crystallized as *ca.* 100–200 nm sized nanoparticles on the surface of TiH_2_. Hydroxide in Ba(OH)_2_ involved BaTiO_2.5_H_0.5_ formation. Ru-loaded catalysts inhibited hydrogen poisoning and showed higher ammonia synthesis activity compared to that of the Ru–Cs/MgO benchmark catalyst. We believe that further investigation of oxyhydride prepared *via* the wet impregnation method accelerates the use of oxyhydride as ammonia synthesis catalysts.

## Author contributions

Yoshihiro Goto: conceptualization, investigation, writing – original draft, Masashi Kikugawa: investigation, Keisuke Kobayashi: data curation, Yuichi Manaka: validation, Tetsuya Nanba: conceptualization, project administration, Hideyuki Matsumoto: formal analysis Mitsuru Matsumoto: validation, Masakazu Aoki: supervision, project administration, Haruo Imagawa: supervision, writing-review &editing.

## Conflicts of interest

There are no conflicts to declare.

## Supplementary Material

RA-013-D3RA01539D-s001
