# Supplementary material for: Facile formation of barium titanium oxyhydride on a titanium hydride surface as an ammonia synthesis catalyst
Source: RSC Adv. 2023 May 22;13(23):15410–5. doi: 10.1039/d3ra01539d (PMC10201397; doi:10.1039/d3ra01539d)
Supplement: RA-013-D3RA01539D-s001 [file RA-013-D3RA01539D-s001.pdf]

Electronic Supplementary Material (ESI) for RSC Advances.
This journal is © The Royal Society of Chemistry 2023

Supplementary Information for

Facile formation of barium titanium oxyhydride on a titanium hydride surface as an ammonia synthesis catalyst

Yoshihiro Goto,[*a] Masashi Kikugawa,[a] Keisuke Kobayashi,[b] Yuichi Manaka,[b] Tetsuya Nanba,[b] Hideyuki Matsumoto,[b,c] Mitsuru Matsumoto,[a] Masakazu Aoki,[a] and Haruo Imagawa[a]

[a] *Toyota Central R&D Labs., Inc., 41-1 Yokomichi, Nagakute, Aichi 480-1192, Japan.*

[b] *Renewable Energy Research Center, National Institute of Advanced Industrial Science and Technology, 2-2-9 Machiikedai, Koriyama, Fukushima 963-0298, Japan.*

[c] *Department of Chemical Science and Engineering, School of Materials and Chemical Technology, Tokyo Institute of Technology, 2-12-1 Ookayama, Meguro-ku, Tokyo, 152–8552, Japan.*

1. Supplementary figures

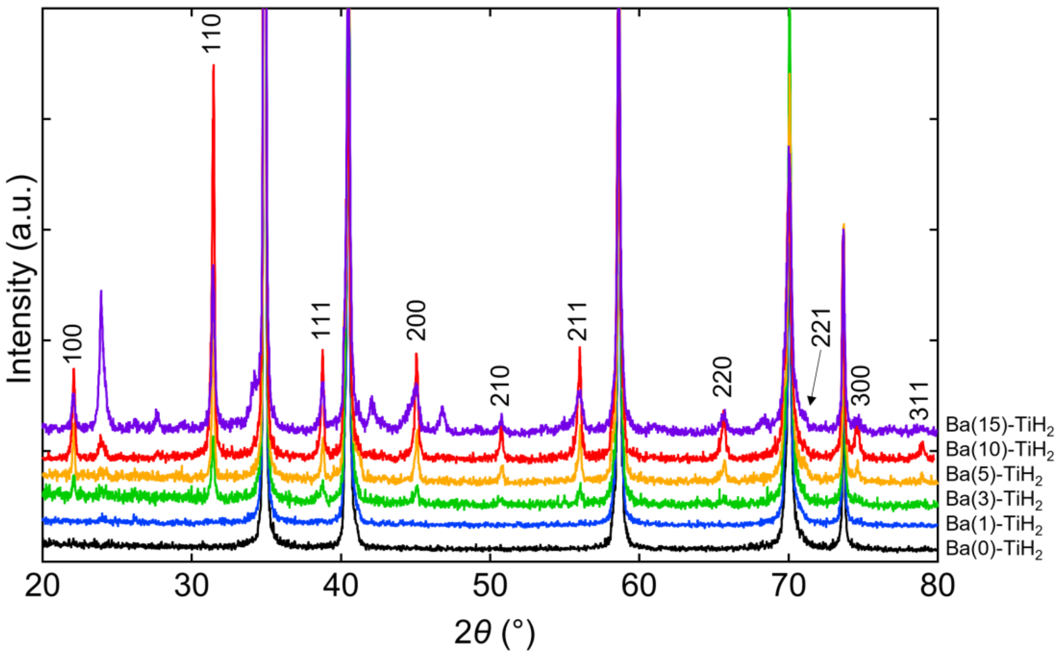

Fig. S1. Expanded XRD patterns of Ba(α)-TiH_2 (α = 0, 1, 3, 5, 10, and 15) with Bragg reflections attributed to $BaTiO_{2.5}H_{0.5}$.

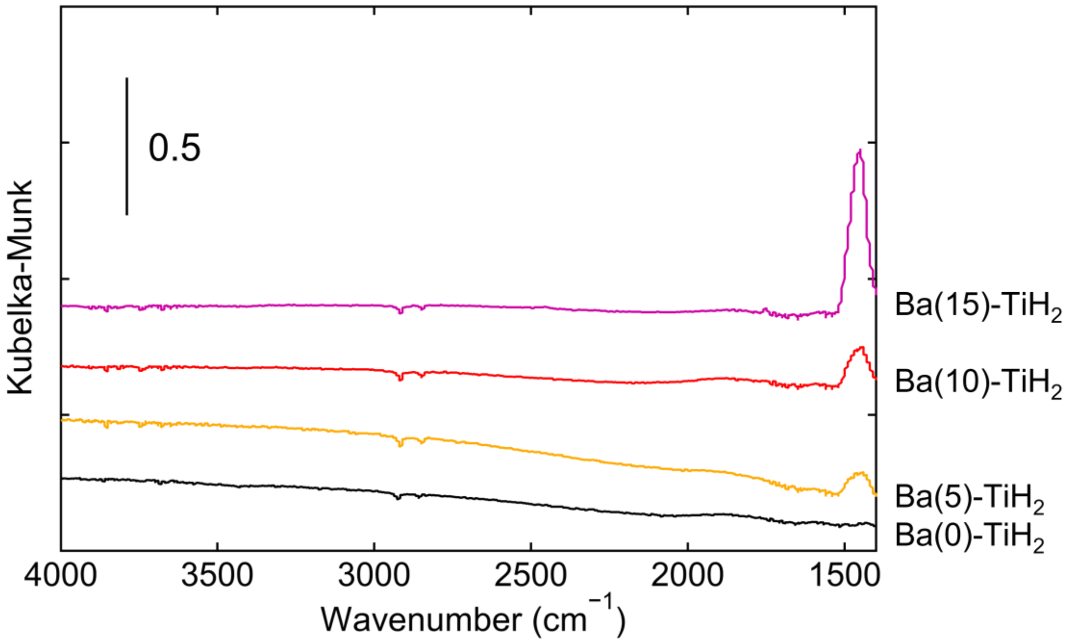

Fig. S2. FT-IR spectra of Ba(α)-TiH_2 (α = 0, 5, 10, and 15) at 50 °C in flowing He. Samples were pretreated at 200 °C for 40 min in flowing He prior to analysis. Peaks at around 1450 cm^{-1} are assigned to the CO_3^{2-} ions of $BaCO_3$.[S1]

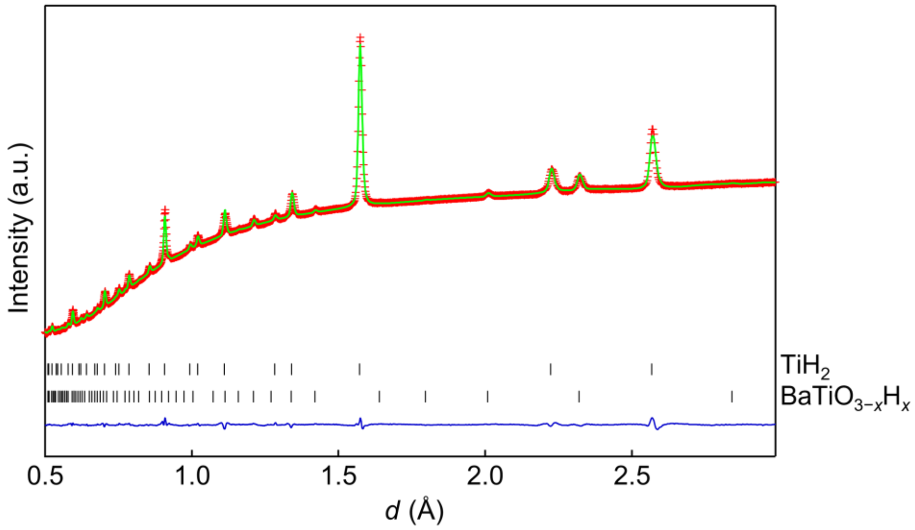

Fig. S3. Rietveld refinement profile for the ND pattern of Ba(10)-TiH_2. The red crosses, green solid line, and blue solid line correspond to observed and calculated intensities and their differences, respectively. The black ticks highlight the positions of the peaks corresponding to TiH_2 and $BaTiO_{3-x}H_x$.

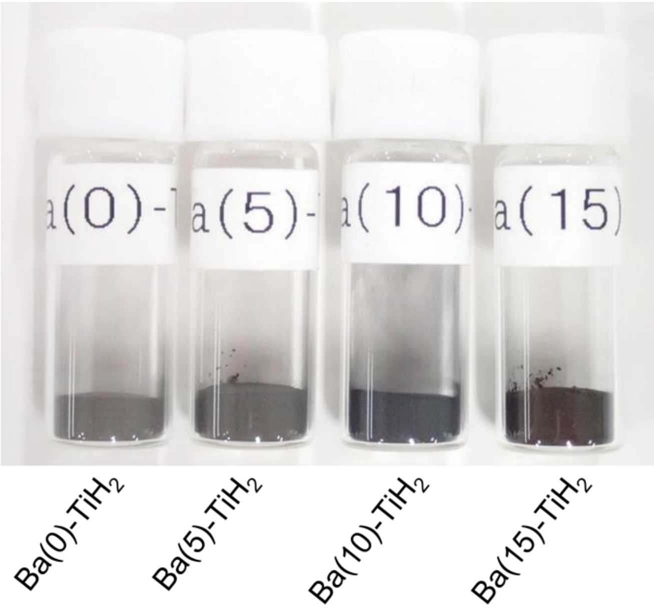

Fig. S4. Color changes accompanying the formation of $BaTiO_{2.5}H_{0.5}$ and $BaCO_3$ on the TiH_2 surface.

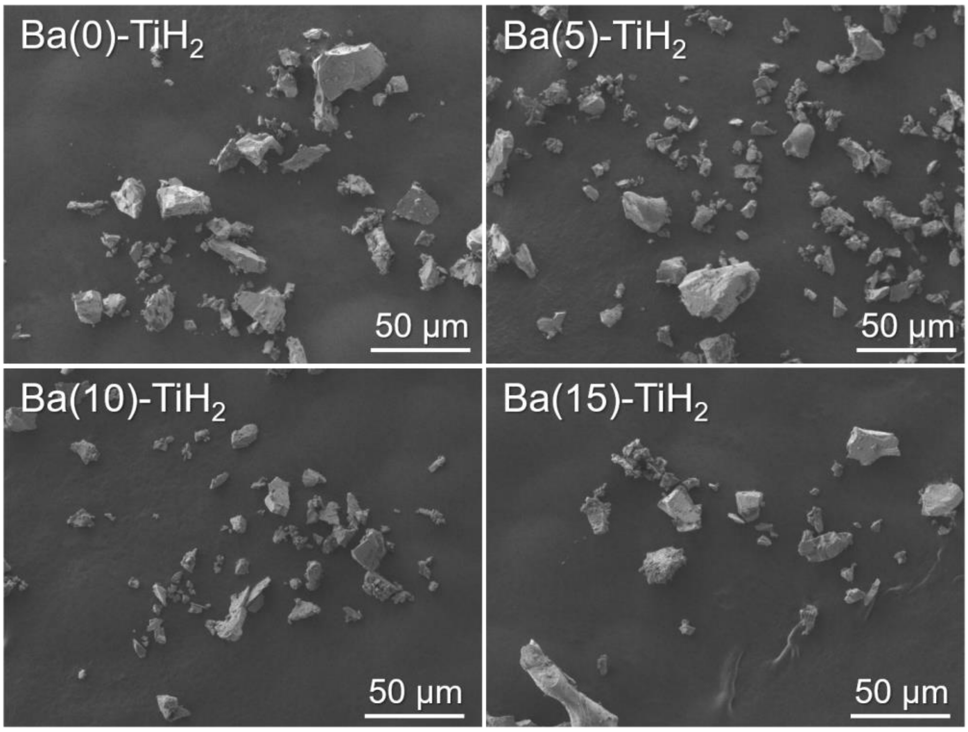

Fig. S5. Scanning electron microscopy (SEM) images of Ba(α)-TiH_2 (α = 0, 5, 10, and 15) at 500× magnification.

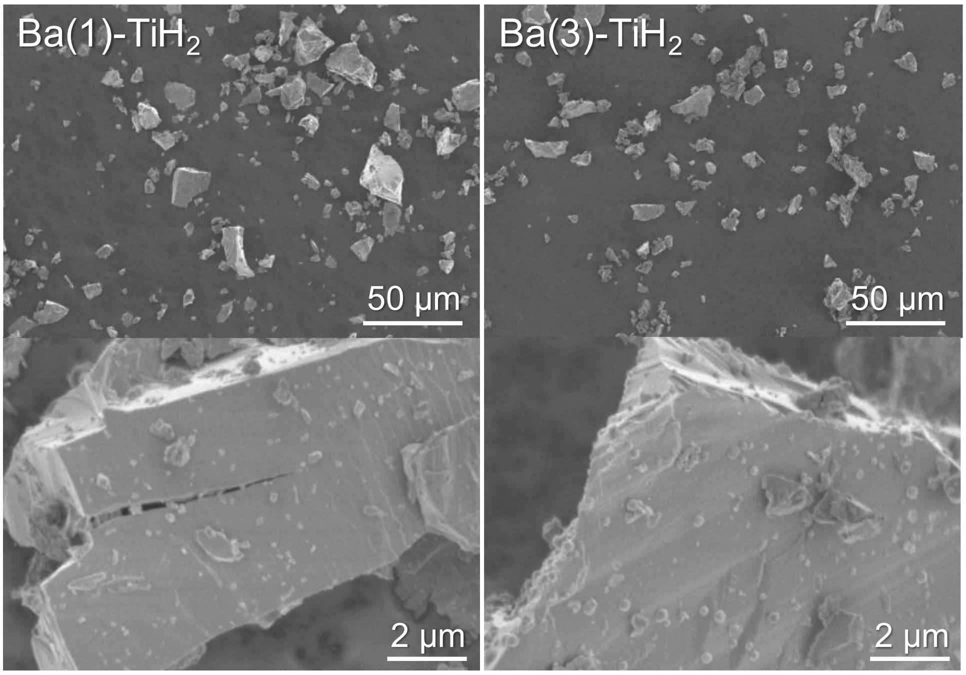

Fig. S6. Scanning electron microscopy (SEM) images of Ba(α)-TiH_2 (α = 1 and 3) at 500× and 10000× magnifications.

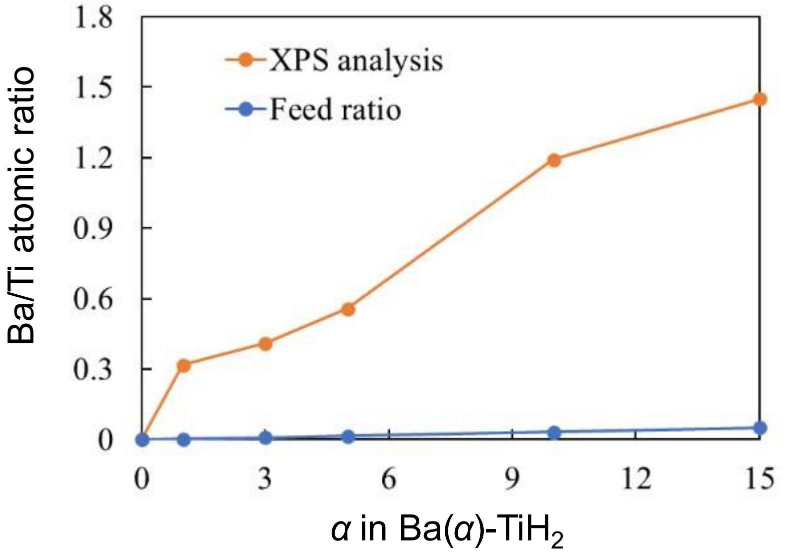

Fig. S7. Ba/Ti atomic ratio of Ba(α)-TiH_2 (α = 0, 1, 3, 5, 10, and 15) estimated from XPS analysis and the feed ratio.

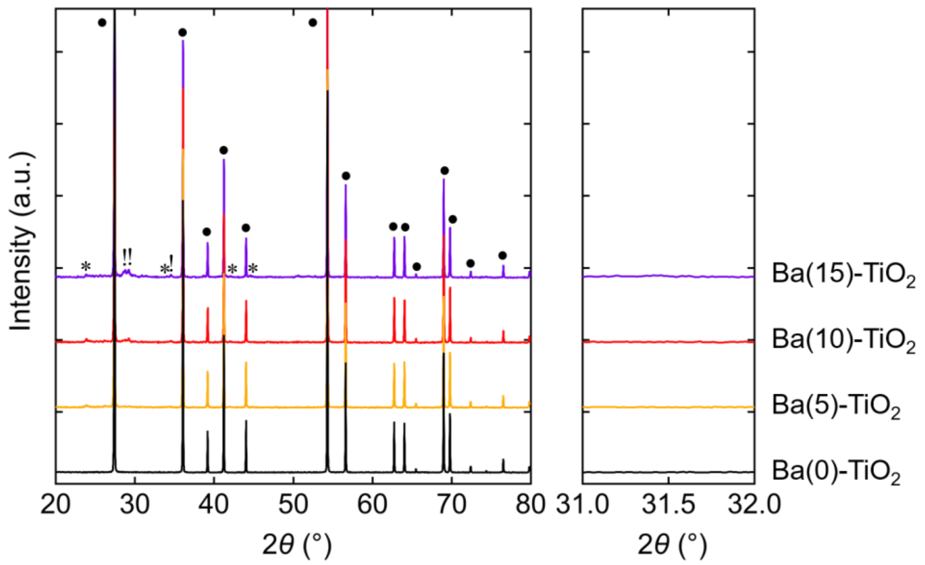

Fig. S8. XRD patterns of Ba(α)-TiO_2 (α = 0, 5, 10, and 15). Circles (●), exclamation marks (!), and asterisks (*) indicate peaks arising from rutile TiO_2, Ba_2TiO_4, and $BaCO_3$, respectively.

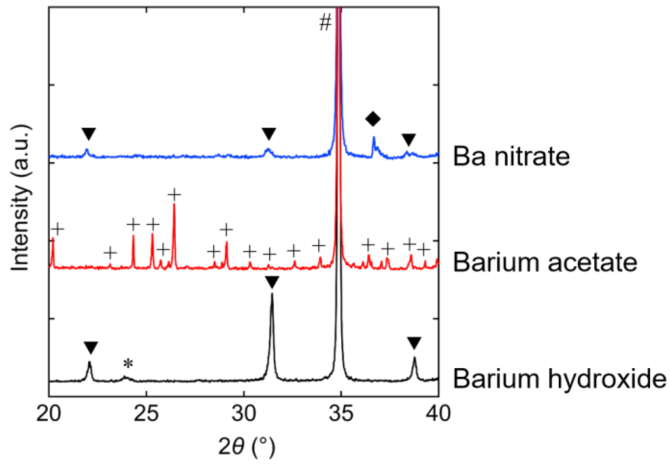

Fig. S9. XRD patterns of Ba(10)-TiH_2 prepared from various barium reagents. Hashtags (#), triangles (▼), asterisks (*), pluses (+), and squares indicate peaks arising from TiH_2, $BaTiO_{2.5}H_{0.5}$, $BaCO_3$, $Ba(CH_3COO)_2$, and $Ba(NO_3)_2$, respectively.

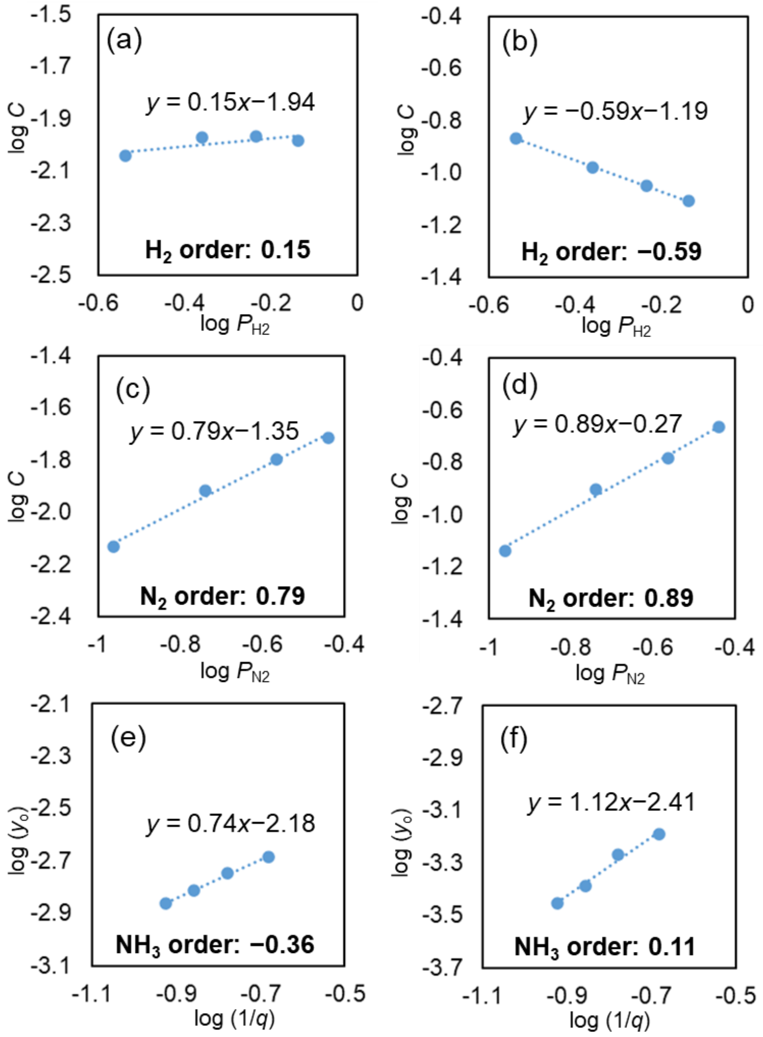

Fig. S10. Dependence of H_2 partial pressure (a, b), N_2 partial pressure (c, d), and flow rate (e, f) on the ammonia synthesis rates of Ru/Ba(10)-TiH_2 (a, c, e) and Ru-Cs/MgO (b, d, f) at 350 °C and 0.1 MPa. The reaction orders of the ammonia synthesis reaction were determined by the method reported by Aika et al.[S2]

3. Supplementary tables

Table S1. Structural data for $BaTiO_{3-x}H_x$[a] in Ba(10)-TiH_2 obtained by the Rietveld refinement of the neutron diffraction pattern.

Atom	Site	*g*	*x*	*y*	*z*	U_{iso} (Å^2)
Ba	1*a*	1	0	0	0	0.0050(6)
Ti	1*b*	1	0.5	0.5	0.5	0.0050(6)
O	3*c*	0.886(2)	0	0.5	0.5	0.0079(5)
H	3*c*	0.114(2)	0	0.5	0.5	0.0079(5)

[a] Space group *Pm*-3*m* (No. 221), a = 4.01659(8) Å, R_{wp} = 0.7173%, R_p = 0.5601%, S = 4.9372.

Table S2. Elemental composition of Ba(α)-TiH_2 (α = 0, 1, 3, 5, 10, and 15) estimated from XPS analysis.

Sample	Ba ($3d_5$)	Ti (2p)	O (1s)
Ba(0)-TiH_2	0	32.21	67.79
Ba(1)-TiH_2	6.99	22.01	71.01
Ba(3)-TiH_2	9.06	22.05	68.88
Ba(5)-TiH_2	11.26	20.23	68.51
Ba(10)-TiH_2	17.87	15.00	67.13
Ba(15)-TiH_2	19.57	13.50	66.93

Table S3. Reaction conditions[a] and ammonia synthesis rates for kinetic analysis.

Reaction order	H_2/N_2 ratio	Flow rate (mL min^{-1})				NH_3 synthesis rate (mmol g^{-1} h^{-1})	
		H_2	N_2	Ar	Total	Ru/Ba(10)-TiH_2	Ru-Cs/MgO
H_2	2	32	16	62	110	0.87	0.24
	3	48	16	46	110	0.98	0.18
	4	64	16	30	110	0.99	0.15
	5	80	16	14	110	0.96	0.13
N_2	5	60	12	38	110	0.75	0.12
	3	60	20	30	110	1.08	0.22
	2	60	30	20	110	1.32	0.30
	1.5	60	40	10	110	1.52	0.41
NH_3	3	48	16	16	80	1.34	0.21
	3	60	20	20	100	1.46	0.22
	3	72	24	24	120	1.50	0.20
	3	84	28	28	140	1.56	0.20

[a] All data were collected at 350 °C and atmospheric pressure.

4. References

S1 E. Roedel, A. Urakawa, S. Kureti and A. Baiker, *Phys. Chem. Chem. Phys.*, 2008, **10**, 6190.

S2 K. Aika, M. Kumasaka, T. Oma, O. Kato, H. Matsuda, N. Watanabe, K. Yamazaki, A. Ozaki and T. Onishi, *Appl. Catal.* 1986, **28**, 57.
